# Icotrokinra, a Targeted Oral Peptide, in Participants With Moderate-to-Severe Plaque Psoriasis and Self-Reported Psoriatic Arthritis: Results From a Pooled Analysis of the Phase 3 ICONIC-LEAD, ICONIC-ADVANCE 1, and ICONIC-ADVANCE 2 Trials

**DOI:** 10.1177/24755303261494526

**Published:** 2026-10-18

**Authors:** Iain B. McInnes, Linda Stein Gold, Robert Bissonnette, Mark G. Lebwohl, Ofelia Reyes-Servin, Joseph Cafone, Ya-Wen Yang, Soumya D. Chakravarty, Charles Iaconangelo, Shu Li, Tasneam Shagroni, Karissa Lozenski, April W. Armstrong, Joseph F. Merola

**Affiliations:** 1 MVLS College Office, Wolfson Medical School Building, College of Medical Veterinary and Life Sciences, University of Glasgow, Glasgow, UK; 2 2971 Henry Ford Health System, West Bloomfield, MI, USA; 3 586953 Innovaderm Research, Montreal, QC, Canada; 4 Department of Dermatology, Icahn School of Medicine at Mount Sinai, New York, NY, USA; 5 Johnson & Johnson, Spring House, PA, USA; 6 Johnson & Johnson, Horsham, PA, USA; 7 Drexel University College of Medicine, Philadelphia, PA, USA; 8 Johnson & Johnson, San Francisco, CA, USA; 9 Department of Dermatology, University of California Los Angeles, Los Angeles, CA, USA; 10 Department of Dermatology, and Department of Medicine, Division of Rheumatology, UT Southwestern Medical Center and O'Donnell School of Public Health, Dallas, TX, USA

**Keywords:** icotrokinra, interleukin-23 inhibition, interleukin-23 receptor, oral peptide, patient-reported outcomes, plaque psoriasis, psoriatic arthritis

## Abstract

**Background:**

Icotrokinra, a targeted oral peptide that precisely blocks the interleukin-23 receptor, is approved to treat adult and pediatric (≥12 years, ≥40 kg) patients with moderate-to-severe plaque psoriasis (PsO).

**Objective:**

Assess psoriatic arthritis (PsA)–specific outcomes through Week (W) 16 in a subgroup of phase-3 study participants with moderate-to-severe plaque PsO and self-reported medical history of PsA (PsO+PsA).

**Methods:**

Post-hoc analyses pooled data from ICONIC-LEAD and ICONIC-ADVANCE 1 and 2 among PsO+PsA participants randomized to once-daily icotrokinra 200 mg or placebo through W16. PsA-specific outcomes included mean change from baseline and ≥50% improvement in Patient’s Global Assessment (PtGA) of PsA disease activity and Patient’s Assessment of PsA Pain, and percent change from baseline in modified Nail Psoriasis Severity Index (mNAPSI). *P* values are nominal.

**Results:**

Analyses included 216 participants (149 icotrokinra, 67 placebo). At W8, icotrokinra-treated participants reported greater improvements vs placebo in PtGA of PsA disease activity (−18.5 vs −4.8; *P* < 0.001) and PsA Pain (−17.1 vs −4.6; *P* < 0.001), with improvement sustained at W16 (−19.2 vs −0.8; *P* < 0.001; −19.5 vs −7.8; *P* = 0.003). At W16, greater proportions of icotrokinra-treated vs placebo-treated participants achieved ≥50% improvement in PtGA of PsA disease activity (47.0% vs 10.7%; *P* < 0.001) and PsA Pain (45.5% vs 16.1%; *P* < 0.001). At W16, icotrokinra improved mNAPSI by 41.6% (vs 118.6% worsening with placebo; *P* < 0.001). Adverse event rates were similar between icotrokinra and placebo.

**Conclusions:**

Icotrokinra demonstrated early, substantial improvements in participant-reported PsA activity and PsA Pain, and nail psoriasis, with a favorable safety profile through W16 in participants with PsO+PsA.

## Introduction

Plaque psoriasis (PsO) and psoriatic arthritis (PsA) are common, chronic, immune-mediated inflammatory diseases that comprise psoriatic disease.^1,2^ PsA coexists in approximately 30% of individuals with PsO.^2,3^ PsA can develop years after PsO onset and involves inflammation across multiple domains (peripheral synovitis, enthesitis, dactylitis, nail and skin involvement, and axial disease), increasing the risk of irreversible joint damage over time.^2,4^ Joint pain, swelling, and stiffness can impair physical function and daily activities, and paired with PsO, can reduce health-related quality of life (HRQoL).^5^ Interleukin (IL)-23–mediated signaling plays a critical role in the inflammation underlying both PsO and PsA, and biologics that target IL-23 have demonstrated robust therapeutic benefits across skin, nail, and joint domains.^6,7^ All currently approved IL-23–targeting biologics are administered subcutaneously,^8-11^ and an unmet need exists for patients with needle aversion or those who prefer oral treatments.^12,13^

Icotrokinra is a targeted oral peptide that blocks the IL-23 receptor with high specificity, and thereby inhibits IL-23 pathway signaling.^14^ Icotrokinra is the first oral therapy with this mechanism of action to gain US Food and Drug Administration approval for the treatment of moderate-to-severe plaque PsO in adult and pediatric (≥12 years, ≥40 kg) patients^15^ and is undergoing evaluation for the treatment of active PsA in parallel phase 3 studies and inflammatory bowel disease in phase 2/3 studies. In the phase 3, double-blind, randomized, placebo-controlled ICONIC-LEAD (ClinicalTrials.gov Identifier: NCT06095115), ICONIC-ADVANCE 1 (NCT06143878), and ICONIC-ADVANCE 2 (NCT06220604) studies in adult and adolescent (ICONIC-LEAD) participants with moderate-to-severe plaque PsO, icotrokinra demonstrated significantly higher rates of skin clearance compared with placebo at Week (W) 16, with a safety profile comparable to placebo.^16,17^ In ICONIC-ADVANCE 1 and 2 in adult participants, icotrokinra demonstrated superior skin clearance rates compared with deucravacitinib at W16 and W24.^17^ These pivotal studies enrolled participants with moderate-to-severe plaque PsO, including those with a self-reported history of PsA, thereby providing an opportunity to evaluate the effects of icotrokinra on skin outcomes and participant-reported outcomes (PROs) relevant to PsA.

Here, we describe post hoc analyses of participants with moderate-to-severe plaque PsO and a self-reported medical history of PsA (PsO+PsA) from ICONIC-LEAD, ICONIC-ADVANCE 1, and ICONIC-ADVANCE 2. These analyses assessed PsA- and PsO-relevant outcomes, HRQoL, and adverse events (AEs) among those receiving icotrokinra or placebo through W16.

## Methods

### Trial Design and Oversight

ICONIC-LEAD, ICONIC-ADVANCE 1, and ICONIC-ADVANCE 2 are ongoing, global, phase 3, randomized, multicenter, double-blind trials with a placebo-controlled period through W16. ICONIC-ADVANCE 1 and 2 also included an active-comparator (deucravacitinib) period through W24. All trials were conducted in accordance with the Declaration of Helsinki and the International Council for Harmonisation Good Clinical Practice guidelines, with the institutional review board or independent ethics committee approval at each site. All participants provided written informed consent before any trial-specific procedures. Study design details and protocols have been published previously.^16,17^

Participants were randomized 2:1 in ICONIC-LEAD and ICONIC-ADVANCE 1, and 4:1 in ICONIC-ADVANCE 2, to receive once-daily oral icotrokinra 200 mg or placebo for 16 weeks. Following W16, all placebo-randomized participants received icotrokinra through W156. These post-hoc pooled analyses focused on the 16-week placebo-controlled period that was common across all 3 trials.

### Participants

Eligible individuals were ≥12 years of age in ICONIC-LEAD or ≥18 years of age in ICONIC-ADVANCE 1 and 2, had a diagnosis of plaque PsO for ≥26 weeks with moderate-to-severe disease (total affected body surface area [BSA] ≥10%, Psoriasis Area and Severity Index [PASI] score ≥12, Investigator’s Global Assessment [IGA] score ≥3), and were eligible for phototherapy or systemic therapy. Adolescents in ICONIC-LEAD were required to weigh ≥40 kg at baseline. Key exclusion criteria included non-plaque PsO, prior suboptimal response or AEs related to IL-23–targeting biologics or tyrosine kinase 2 inhibitors (ICONIC-ADVANCE studies). Participants included in the pooled post-hoc analyses had a self-reported medical history of PsA (PsO+PsA).

### Assessments

Participants with PsO+PsA completed the Patient’s Global Assessment (PtGA) of PsA disease activity and the Patient’s Assessment of PsA Pain (PsA Patient Pain) using a visual analog scale (PtGA, range, 0 [very poor] to 100 [very well]^18^; Patient Pain, range, 0 [no pain] to 100 [worst possible pain]^19^).

Overall skin and high-impact site PsO extent and severity were assessed by investigators using IGA and PASI. The IGA evaluates plaque PsO severity on a 5-point scale (0 [clear] to 4 [severe]).^20^ The PASI quantifies PsO extent and severity on a scale from 0 to 72, with higher scores indicating more extensive involvement and greater severity.^21^ Nail PsO severity was also assessed by investigators using the modified Nail Psoriasis Severity Index (mNAPSI) on a scale of 0 to 130, with higher scores indicating greater severity.^22^ High-impact site PsO clearance was also evaluated using the Fingernail Physician’s Global Assessment (f-PGA; 0 [clear] to 4 [severe]),^23^ scalp-specific IGA (ss-IGA; 0 [absence of disease] to 4 [severe disease]), static Physician’s Global Assessment of Genitalia (sPGA-G; 0 [clear] to 5 [very severe]),^24^ and Physician’s Global Assessment of hands and feet (hf-PGA; 0 [clear] to 4 [severe]).^25^

Participants reported PsO symptoms using the Psoriasis Symptoms and Signs Diary (PSSD), which includes 11 items.^26^ Each item is graded from 0 (absent) to 10 (worst imaginable); PSSD symptom score ranges from 0 to 100, with higher scores indicating worse symptoms. An item-level score for PSSD itch ranges from 0 to 10, with higher scores indicating more severe disease. Adult participants completed the Genital Psoriasis Sexual Frequency Questionnaire (GenPs-SFQ)^27^ Item 2, which asks how often genital PsO symptoms have limited the frequency of sexual activity in the previous 7 days (0 [never] to 4 [always]). HRQoL was assessed at baseline and W16 using the Dermatology Life Quality Index (DLQI), scored from 0 to 30, where lower scores indicate less impact.^28^

### Outcomes

PsA-specific PROs included change from baseline at W8 and W16 and achievement of ≥50% improvement from baseline at W16 on PtGA of PsA disease activity and PsA Patient Pain.

Overall skin clearance outcomes at W16 included achievement of IGA 0/1 (score of 0 [clear] or 1 [minimal] with ≥2-grade improvement from baseline), IGA 0 (score of 0), PASI 90 (≥90% improvement from baseline), and PASI 100 (100% improvement from baseline).

Percent improvement from baseline in mNAPSI score at W16 was evaluated in participants with baseline mNAPSI score >0. Clearance of other high-impact site PsO was evaluated only in participants with baseline scores ≥2 on the site-specific assessment. Outcomes at W16 included achievement of f-PGA score of 0/1 (clear/minimal), f-PGA 0 (score of 0), ss-IGA 0/1 (score of 0 [absence] or 1 [very mild] with ≥2-grade improvement from baseline), ss-IGA 0 (score of 0), sPGA-G 0/1 (score of 0 [clear] or 1 [minimal] with ≥2-grade improvement from baseline), sPGA-G 0 (score of 0), hf-PGA 0/1 (score of 0 [clear] or 1 [almost clear] with ≥2-grade improvement from baseline), and hf-PGA 0 (score of 0).

PsO-specific PROs at W16 included achievement of a PSSD symptom score of 0 among participants with a baseline score >0 and a clinically meaningful improvement (CMI) in itch, defined as a ≥4-point decrease from baseline in PSSD itch score among participants with a baseline score ≥4. Achievement of a GenPs-SFQ Item 2 score of 0 or 1 (never/rarely) at W16 was evaluated among adult participants with baseline GenPs-SFQ Item 2 scores ≥2 and sPGA-G ≥2. Achievement of DLQI 0/1 (no impact of PsO on HRQoL) at W16 was evaluated among those with a baseline DLQI score >1.

### Statistical Analysis

Efficacy analyses included all randomized participants, excluding 3 participants from ICONIC-ADVANCE 2 due to noncompliance with Good Clinical Practice at 1 study site, with evaluable data. Safety analyses included all randomized participants who received ≥1 dose of study drug. Data for participants who discontinued the study drug due to lack of efficacy or AEs of worsening PsO, or who initiated prohibited medication that could affect PsO, were imputed as having no improvement (continuous outcomes) or as nonresponders (binary outcomes). Observed data were used for participants who discontinued study drug for other reasons. After accounting for these intercurrent events, participants with missing data were considered nonresponders for binary outcomes; missing data were not imputed and were accounted for through a mixed-effect model for repeated measures for analyses of continuous outcomes.

For continuous outcomes, least squares mean (LSM) differences and associated *P* values were estimated using a mixed-effect model for repeated measures, with treatment group, visit, study, baseline score, baseline score-by-visit interaction, and treatment group-by-visit interaction as covariates. For binary outcomes, treatment differences and 95% confidence intervals (CIs) were calculated using the Miettinen-Nurminen method with Mantel-Haenszel weights, adjusted as appropriate by study, weight category (adults), and geographic region, or using an exact method, as appropriate. All *P* values are nominal, and AEs were summarized descriptively through W16.

## Results

### Baseline Characteristics

Among the 1555 participants with moderate-to-severe plaque PsO randomized to icotrokinra or placebo across the 3 studies, 216 adults and adolescents had a self-reported medical history of PsA (icotrokinra: 149; placebo: 67), representing 13.9% of the overall pooled study population for these 2 treatment groups. Through W16, 7 (4.7%) participants in the icotrokinra group and 9 (13.4%) in the placebo group discontinued treatment. Discontinuations occurred most commonly due to AEs (icotrokinra: 3 [2.0%]; placebo: 5 [7.5%]), lack of efficacy (icotrokinra: 1 [0.7%]; placebo: 3 [4.5%]), withdrawal by participant (icotrokinra: 2 [1.3%]; placebo: 1 [1.5%]), and loss to follow-up (icotrokinra: 1 [0.7%]; placebo: 0).

Baseline characteristics were generally similar between groups (Table 1). The mean age of participants was 49.5 years (range: 16-79); the majority were male (61.6%) and White (78.7%). Baseline mean duration of PsO was 19.5 years (SD: 12.6), with a mean PASI score of 19.8 (SD: 7.1) and mean affected BSA of 25.2% (SD: 14.5). Baseline mean PtGA of PsA disease activity was 48.6 (SD: 27.0) and mean PsA Patient Pain was 50.5 (SD: 28.0). Nail PsO at baseline was characterized by a mean mNAPSI score of 21.2 (SD: 18.8) among participants with f-PGA >0. Overall, 40.9% of participants had f-PGA ≥2, 86.0% had ss-IGA ≥2, 39.1% had sPGA-G ≥2, and 45.6% had hf-PGA ≥2. Baseline mean DLQI score was 13.6 (SD: 7.4). In total, 80.1% of participants had received prior systemic therapy and 45.8% had received prior biologic therapy.

**Table 1.** Baseline Demographics and Disease Characteristics for the Pooled Subgroup of Participants With PsO and a Self-Reported Medical History of PsA

Characteristic	Icotrokinra (N = 149)	Placebo (N = 67)	Total (N = 216)
**Demographic characteristics**			
Age, years, mean (SD)	49.6 (12.8)	49.1 (13.1)	49.5 (12.8)
Male sex, n (%)	96 (64.4)	37 (55.2)	133 (61.6)
Race, n (%)			
White	114 (76.5)	56 (83.6)	170 (78.7)
Asian	25 (16.8)	9 (13.4)	34 (15.7)
Black or African American	4 (2.7)	2 (3.0)	6 (2.8)
Hispanic or Latino, n (%)	21 (14.1)	17 (25.4)	38 (17.6)
Weight, kg, mean (SD)	91.9 (24.2)	86.4 (21.9)	90.2 (23.6)
BMI, kg/m^2^, mean (SD)	31.3 (7.5)	30.4 (7.6)	31.0 (7.5)
**Disease characteristics**			
PsO duration, years, mean (SD)	19.1 (12.0)	20.4 (13.8)	19.5 (12.6)
IGA score, n (%)			
Severe (4)	25 (16.8)	20 (29.9)	45 (20.8)
Moderate (3)	124 (83.2)	47 (70.1)	171 (79.2)
Mild (2)	0	0	0
PASI score (0-72), mean (SD)	19.4 (6.5)	20.6 (8.2)	19.8 (7.1)
% BSA, mean (SD)	24.9 (14.3)	26.0 (15.0)	25.2 (14.5)

**PsA PROs**	N = 136	N = 57	N = 193
PtGA of PsA disease activity score (0-100), mean (SD)	48.0 (27.0)	50.1 (27.1)	48.6 (27.0)
PsA patient pain score (0-100), mean (SD)	49.3 (27.6)	53.4 (28.7)	50.5 (28.0)
**PsO ** **clinician-** **reported** ** outcomes**			

​	N = 84	N = 33	N = 117
mNAPSI score (0-130), mean (SD)^a^	21.0 (18.6)	21.5 (19.5)	21.2 (18.8)

​	N = 148	N = 67	N = 215
f-PGA score, n (%)			
Severe (4)	5 (3.4)	1 (1.5)	6 (2.8)
Moderate (3)	26 (17.6)	12 (17.9)	38 (17.7)
Mild (2)	35 (23.6)	9 (13.4)	44 (20.5)
ss-IGA score, n (%)			
Severe (4)	23 (15.5)	20 (30.0)	43 (20.0)
Moderate (3)	90 (60.8)	28 (41.8)	118 (54.9)
Mild (2)	15 (10.1)	9 (13.4)	24 (11.2)
sPGA-G score, n (%)			
Very severe (5)	2 (1.4)	0	2 (0.9)
Severe (4)	13 (8.8)	10 (14.9)	23 (10.7)
Moderate (3)	30 (20.3)	11 (16.4)	41 (19.1)
Mild (2)	15 (10.1)	3 (4.5)	18 (8.4)
hf-PGA score, n (%)			
Severe (4)	5 (3.4)	4 (6.0)	9 (4.2)
Moderate (3)	40 (27.0)	12 (17.9)	52 (24.2)
Mild (2)	25 (16.9)	12 (17.9)	37 (17.2)
**PsO PROs**			

​	N = 138	N = 59	N = 197
PSSD symptom score (0-100), mean (SD)	57.7 (25.1)	59.5 (27.1)	58.2 (25.7)
PSSD sign score (0-100), mean (SD)	61.0 (21.1)	61.2 (24.7)	61.1 (22.2)

GenPs-SFQ item 2 score, n (%)	N = 148	N = 65	N = 213
Always (4)	12 (8.1)	8 (12.3)	20 (9.4)
Often (3)	22 (14.9)	3 (4.6)	25 (11.7)
Sometimes (2)	20 (13.5)	7 (10.8)	27 (12.7)

​	N = 148	N = 65	N = 213
DLQI score (0-30), mean (SD)	13.3 (7.2)	14.2 (7.7)	13.6 (7.4)

​	N = 149	N = 67	N = 216
DLQI score >1, n (%)	148 (99.3)	64 (95.5)	212 (98.1)
**Previous PsO medications**			
Systemic therapy^b^	119 (79.9)	54 (80.6)	173 (80.1)
Conventional nonbiologic systemic therapy^c^	78 (52.3)	39 (58.2)	117 (54.2)
Biologic therapy^d^	68 (45.6)	31 (46.3)	99 (45.8)
Phototherapy^e^	44 (29.5)	22 (32.8)	66 (30.6)
Novel nonbiologic therapy^f^	22 (14.8)	6 (9.0)	28 (13.0)

BMI, body mass index; BSA, body surface area; DLQI, dermatology life quality index; f-PGA, Fingernail Physician’s Global Assessment; GenPs-SFQ, Genital Psoriasis Sexual Frequency Questionnaire; hf-PGA, Physician’s Global Assessment of hands and feet; IGA, Investigator’s Global Assessment; mNAPSI, modified Nail Psoriasis Severity Index; PASI, Psoriasis Area and Severity Index; PsA, psoriatic arthritis; PRO, participant-reported outcome; PsO, psoriasis; PSSD, Psoriasis Symptoms and Signs Diary; PtGA, Patient’s Global Assessment; SD, standard deviation; sPGA-G, static Physician’s Global Assessment of Genitalia; ss-IGA, scalp-specific Investigator’s Global Assessment.

The analyzed population is the safety analysis set, including all randomized participants who received ≥1 dose of study drug.

^a^mNAPSI total score is based on participants with an f-PGA score >0.

^b^Includes biologic therapy, conventional nonbiologic systemic therapies, novel (oral) nonbiologic systemic therapies, phototherapy, and 1,25-dihydoxyvitamin D and analogues.

^c^Includes acitretin, azathioprine, cyclosporine, fumarate, methotrexate, and psoralen plus ultraviolet A.

^d^Includes adalimumab, alefacept, briakinumab, brodalumab, certolizumab pegol, efalizumab, etanercept, guselkumab, infliximab, ixekizumab, natalizumab, risankizumab, secukinumab, tildrakizumab, and ustekinumab.

^e^Includes psoralen plus ultraviolet A and ultraviolet B.

^f^Includes apremilast, deucravacitinib, and tofacitinib.

### Psoriatic Arthritis–Specific Participant-Reported Outcomes

At first follow-up for PtGA of PsA disease activity (W8), icotrokinra-treated participants reported greater improvements compared with placebo in PtGA of PsA disease activity, with LSM change from baseline of −18.5 vs −4.8 (nominal *P* < 0.001; Figure 1A). These improvements were sustained in icotrokinra-treated participants through W16, whereas placebo-treated participants reported no improvement or worsening disease (LSM change: −19.2 vs −0.8; nominal *P* < 0.001). A higher proportion of icotrokinra-treated participants reported ≥50% improvement from baseline in PtGA of PsA disease activity (47.0% vs placebo 10.7%; nominal *P* < 0.001; Figure 1B).

**Figure 1.**
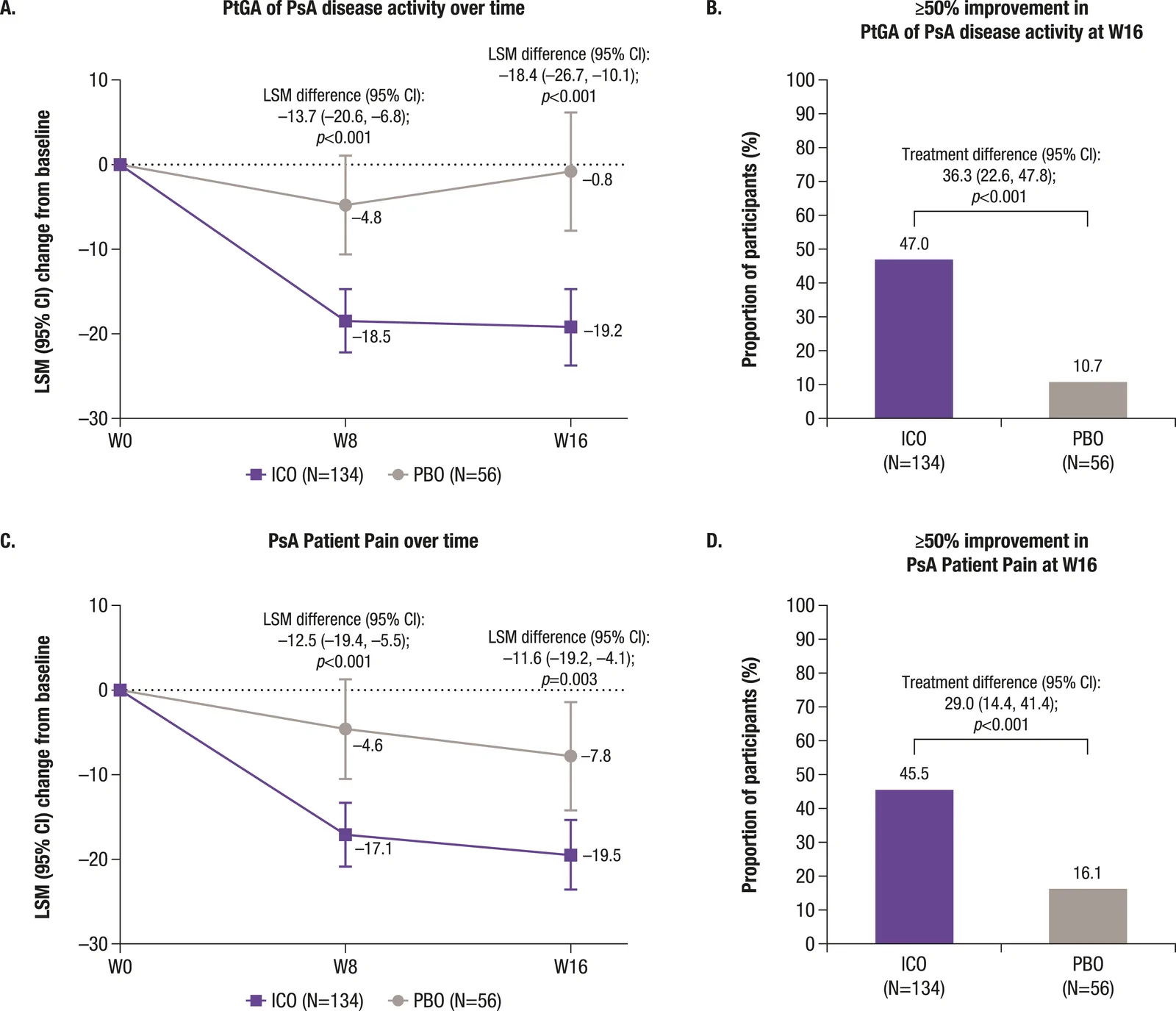
PsA–specific PROs^a^: (A) LSM change from baseline at W8 and W16 in PtGA of PsA disease activity, (B) proportion of participants achieving ≥50% improvement in PtGA of PsA disease activity, (C) LSM change from baseline at W8 and W16 in PsA Patient Pain, and (D) proportion of participants achieving ≥50% improvement in PsA Patient Pain. CI, confidence interval; ICO, icotrokinra; LSM, least squares mean; PBO, placebo; PRO, participant-reported outcome; PsA, psoriatic arthritis; PtGA, Patient’s Global Assessment; W, week. All *P* values are nominal. ^a^Among participants with baseline PtGA of PsA disease activity and PsA Pain

Similarly, for PsA Patient Pain, icotrokinra demonstrated greater improvement from baseline at W8 (LSM change: −17.1 vs −4.6; nominal *P* < 0.001; Figure 1C). These improvements persisted through W16 (LSM change: −19.5 vs −7.8; nominal *P* = 0.003). A greater proportion of icotrokinra-treated vs placebo-treated participants reported ≥50% improvement from baseline in PsA Patient Pain (45.5% vs 16.1%; nominal *P* < 0.001; Figure 1D).

### Psoriasis Outcomes

#### Overall Psoriasis

For overall skin clearance at W16, higher proportions of participants receiving icotrokinra compared with placebo achieved IGA 0/1 (56.5% vs 10.6%; nominal *P* < 0.001) and PASI 90 (45.6% vs 3.0%; nominal *P* < 0.001) responses (Figure 2A). Icotrokinra provided complete skin clearance in 27.9% (IGA 0) and 25.2% (PASI 100) of participants (vs 0% with placebo; both *P* < 0.001; Figure 2B).

**Figure 2.**
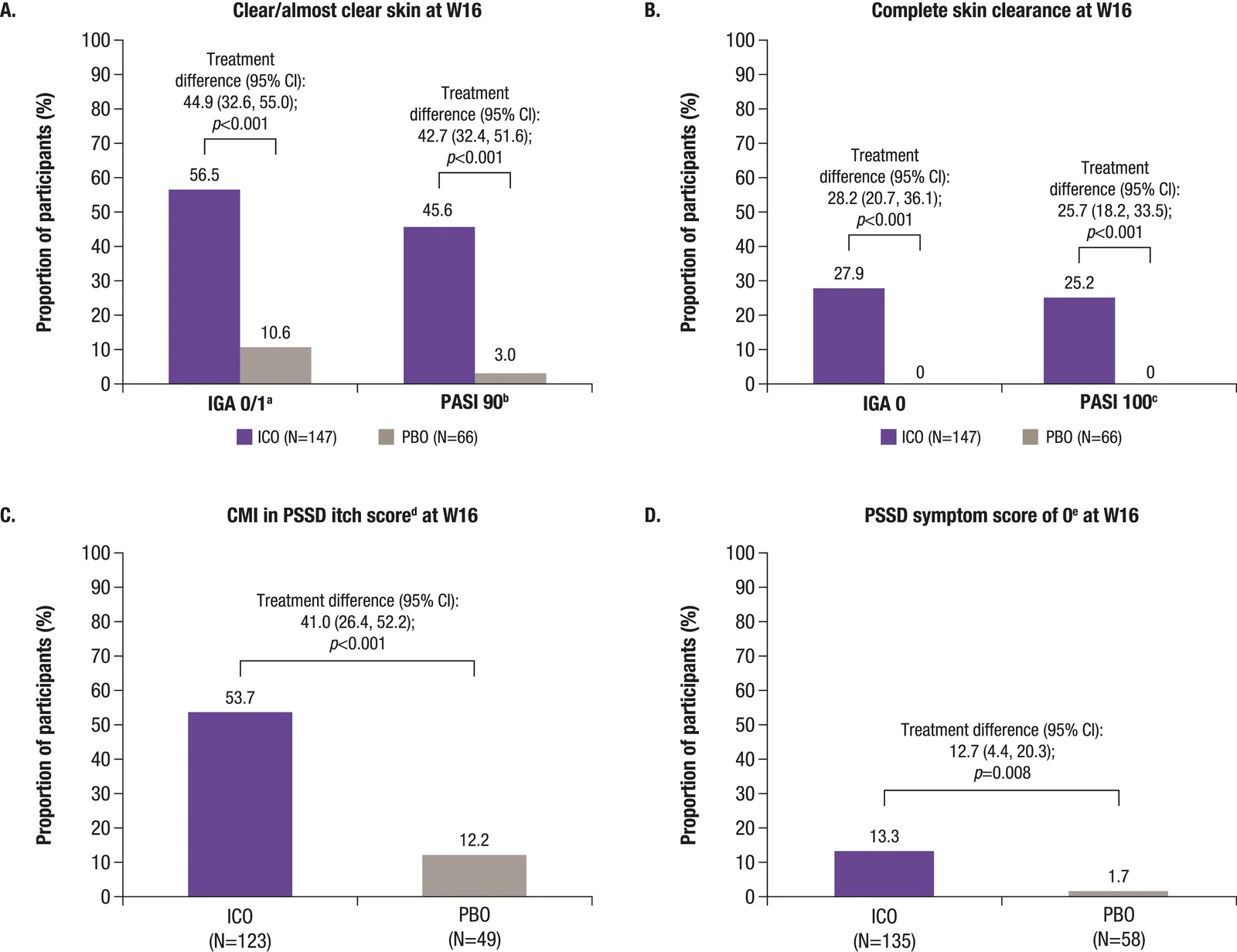
Overall skin clearance and symptom improvements at W16: proportion of participants achieving (A) clear/almost clear skin and (B) complete skin clearance, (C) CMI in PSSD itch score, and (D) PSSD symptom score of 0. CI, confidence interval; CMI, clinically meaningful improvement; ICO, icotrokinra; IGA, Investigator’s Global Assessment; PASI, Psoriasis Area and Severity Index; PBO, placebo; PSSD, Psoriasis Symptoms and Signs Diary; W, week. All *P* values are nominal. ^a^IGA 0/1 is defined as a score of 0/1 with ≥2-grade improvement from baseline. ^b^Defined as ≥90% improvement in PASI score from baseline. ^c^Defined as 100% improvement in PASI score from baseline. ^d^Among participants with baseline PSSD itch score ≥4. ^e^Among participants with baseline PSSD symptom score >0

Among participants with baseline score ≥4, a greater proportion of icotrokinra-treated vs placebo-treated participants reported CMI in PSSD itch score at W16 (53.7% vs 12.2%; nominal *P* < 0.001) (Figure 2C). A greater proportion of icotrokinra-treated vs placebo-treated participants also reported resolution of PsO symptoms (PSSD symptom score of 0) at W16 among participants with baseline score >0 (13.3% vs 1.7%; nominal *P* = 0.008; Figure 2D).

#### High-Impact Site Psoriasis

Among participants with nail PsO (baseline mNAPSI >0), icotrokinra-treated participants demonstrated marked improvement in mNAPSI from baseline to W16 (LSM percent change: −41.6), while those receiving placebo exhibited worsening disease (LSM percent change: +118.6; nominal *P* < 0.001; Figure 3A). For participants with fingernail involvement (f-PGA ≥2), greater proportions of icotrokinra-treated vs placebo-treated participants achieved f-PGA 0/1 (46.9% vs 9.5%; nominal *P* = 0.005) and f-PGA 0 (18.8% vs 9.5%; nominal *P* = 0.827; Figure 3B).

**Figure 3.**
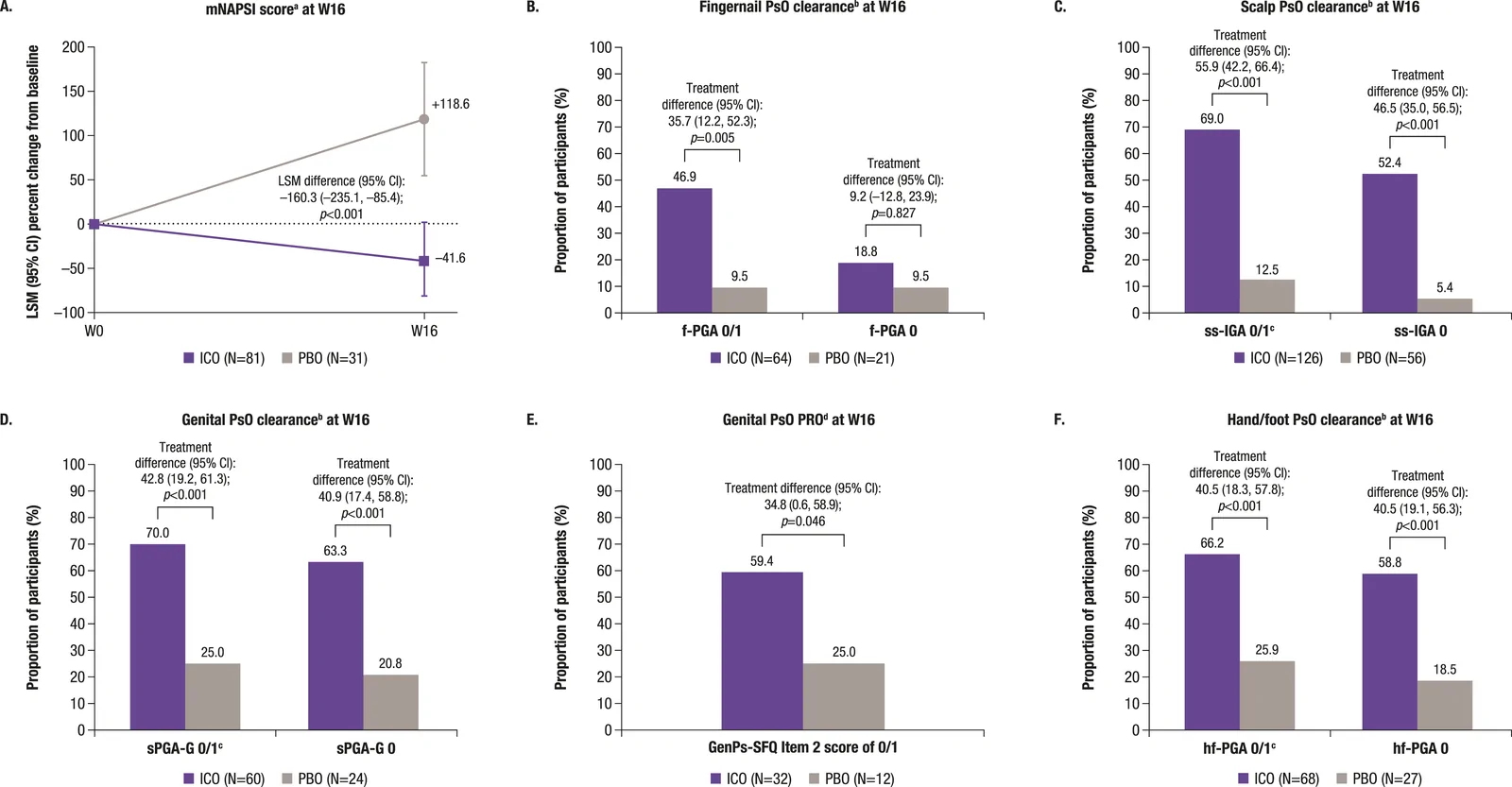
High-impact site PsO clearance and symptom improvements at W16: (A) percent change from baseline in mNAPSI score; proportion of participants achieving absent/clear or minimal/almost clear and completely absent/clear in high-impact sites: (B) f-PGA, (C) ss-IGA, (D) sPGA-G, (F) hf-PGA; and proportion of participants achieving (E) GenPs-SFQ Item 2 score of 0 or 1. CI, confidence interval; f-PGA, Fingernail Physician’s Global Assessment; GenPs-SFQ, Genital Psoriasis Sexual Frequency Questionnaire; hf-PGA, Physician’s Global Assessment of hands and feet; ICO, icotrokinra; LSM, least squares mean; mNAPSI, modified Nail Psoriasis Severity Index; PBO, placebo; PRO, participant-reported outcome; PsO, psoriasis; sPGA-G, static Physician’s Global Assessment of Genitalia; ss-IGA, scalp-specific Investigator’s Global Assessment; W, week. All *P* values are nominal. ^a^Among participants with baseline mNAPSI score >0. ^b^Among participants with baseline scores at each high-impact site ≥2. ^c^ss-IGA 0/1, sPGA-G 0/1, and hf-PGA 0/1 responses are defined as a score of 0/1 with ≥2-grade improvement from baseline. ^d^Among participants with baseline GenPs-SFQ Item 2 score ≥2 and sPGA-G ≥2

Among those with at least mild scalp involvement (ss-IGA ≥2), greater proportions of participants treated with icotrokinra vs placebo achieved ss-IGA 0/1 (69.0% vs 12.5%; nominal *P* < 0.001) and ss-IGA 0 (52.4% vs 5.4%; nominal *P* < 0.001; Figure 3C). Among participants with at least mild genital involvement (s-PGA-G ≥2), greater proportions of participants receiving icotrokinra vs placebo achieved s-PGA-G 0/1 (70.0% vs 25.0%; nominal *P* < 0.001) and s-PGA-G 0 (63.3% vs 20.8%; nominal *P* < 0.001; Figure 3D). In participants with baseline genital PsO symptom burden (GenPs-SFQ Item 2 score ≥2 and sPGA-G ≥2), 59.4% of those treated with icotrokinra reported no or rare impact of genital PsO on sexual activity (GenPs-SFQ Item 2 score 0/1) compared with 25.0% receiving placebo (nominal *P* = 0.046; Figure 3E). Among those with at least mild involvement of the hands and/or feet (hf-PGA ≥2), greater proportions of participants treated with icotrokinra vs placebo achieved hf-PGA 0/1 (66.2% vs 25.9%; nominal *P* < 0.001) and hf-PGA 0 (58.8% vs 18.5%; nominal *P* < 0.001; Figure 3F).

### Health-Related Quality of Life

Among participants with DLQI >1, a greater proportion of participants receiving icotrokinra vs placebo reported no impact of PsO on HRQoL (DLQI 0/1: 47.3% vs 11.1%; nominal *P* < 0.001) at W16.

### Adverse Events

Through W16, AE rates within the PsO+PsA cohort were comparable between the icotrokinra (49.3% [73/148]) and placebo (50.7% [34/67]) groups; mean follow-up duration was 15.8 and 15.3 weeks, respectively. Infection was reported in 18.9% (28/148) of icotrokinra-treated participants and 25.4% (17/67) of placebo-treated participants. The most common infection (≥5% in any groups) was upper respiratory infection (icotrokinra: 4.7% [7/148]; placebo: 3.0% [2/67]). Gastrointestinal AEs were comparable between groups (icotrokinra: 8.1% [12/148]; placebo: 6.0% [4/67]).

## Discussion

The present pooled analysis extends the findings from the ICONIC-LEAD and ICONIC-ADVANCE 1 and 2 studies^16,17^ by providing preliminary evidence that icotrokinra can achieve early and substantial improvements in PsA-specific symptoms in adults and adolescents with moderate-to-severe plaque PsO and a self-reported medical history of PsA.

Management of PsA requires addressing multiple domains, including skin, peripheral and axial joints, enthesitis, dactylitis, and nails.^29^ Icotrokinra-treated participants demonstrated substantial improvements in PtGA of PsA disease activity and in PsA Patient Pain by W8 (first follow-up for PtGA of PsA disease activity and PsA Patient Pain), with nearly one-half achieving ≥50% improvement in these outcomes at W16. The criteria for ≥50% improvement in PtGA of PsA disease activity and PsA Patient Pain are 2 components of the American College of Rheumatology 50% (ACR50) response criteria, a stringent, clinically relevant measure of improvement in PsA, supporting the clinical relevance of these participant-reported improvements.^30,31^ Nail PsO is also associated with an increased risk of developing PsA and worse disease outcomes, is particularly recalcitrant to therapy, and disproportionately affects HRQoL.^32,33^ Accordingly, nail involvement is considered as a clinical feature warranting timely assessment and treatment initiation or escalation in PsA.^34,35^ At W16, icotrokinra substantially reduced nail PsO severity from baseline as assessed by mNAPSI and demonstrated achievement of clear or minimal nail disease in almost half of participants with baseline nail involvement as assessed by f-PGA, which are notable findings given the slow growth of nails and their predictive role in PsA.^32,33^ The ongoing ICONIC-PsA 1 and 2 studies (NCT06878404, NCT06807424) will elucidate the effect of icotrokinra on multidomain outcome measures (including joint, nail, and skin outcomes) in PsA.

Patients with PsO and PsA frequently exhibit reduced skin clearance response to therapy and substantial residual disease burden compared with those who have PsO alone.^36,37^ PsO involving high-impact sites is difficult to treat, may cause disability, and have considerable impacts on HRQoL.^38,39^ In this PsO+PsA subgroup, icotrokinra demonstrated high rates of both overall and high-impact site skin clearance and PsO symptom resolution, including highly burdensome itch, and reduced impact of PsO on HRQoL, consistent with observations in the overall PsO study populations.^16,17^ Together, these participant- and clinician-reported improvements in PsA and PsO support a comprehensive clinical benefit of icotrokinra in multidomain disease burden for patients with PsO+PsA.

Participant retention rates in this pooled PsO+PsA population were similar to those in the overall PsO study populations.^16,17^ Baseline characteristics were generally consistent with those reported in dedicated PsA trials, including baseline PtGA and mNAPSI scores.^40-45^ This suggests that the benefits observed here may be relevant to the broader PsA population, although confirmation in dedicated PsA trials will be important.

Through W16, safety findings in this analysis were consistent with those in the overall PsO trial population and in other studies of icotrokinra,^16,17,46,47^ with comparable AE rates between icotrokinra and placebo and no safety signals emerging. These findings demonstrate that icotrokinra’s safety profile in participants with PsO+PsA aligns closely with that observed in the broader PsO population.^15^

This analysis is limited by reliance on self-reported medical history of PsA rather than formal classification using the Classification Criteria for Psoriatic Arthritis (CASPAR) criteria, and using PROs for assessing PsA disease activity. However, these PROs are important in PsA assessment because they capture the patient’s perception of disease severity, symptoms, functional limitations, and HRQoL, factors that often reflect disease progression, influence treatment satisfaction, and may affect long-term outcomes.^48^ Potential unblinding due to visible skin response is also an inherent limitation of PsA-related PROs reported in this analysis. The 16-week observation period reflects only early treatment effects. Although durability of these early benefits requires further assessment, these findings suggest that patients with PsA may experience prompt, clinically meaningful pain relief and improvement in patient-perceived PsA disease activity. Ongoing phase 3, multicenter, randomized, double-blind, placebo-controlled trials in biologic-naïve and biologic-experienced participants with active PsA will further inform the efficacy and safety of icotrokinra in this population.

## Conclusion

Through 16 weeks, pooled data from ICONIC-LEAD, ICONIC-ADVANCE 1, and ICONIC-ADVANCE 2 demonstrate that icotrokinra-treated adults and adolescents with moderate-to-severe plaque PsO and a self-reported medical history of PsA experienced early and substantial improvements in PtGA of PsA disease activity and PsA Patient Pain and marked improvement in nail disease. Consistent with outcomes observed in the overall plaque PsO study populations, this cohort demonstrated high rates of overall and high-impact site skin clearance and enhanced HRQoL, with a safety profile comparable to placebo through W16. Together with prior evidence,^16,17,46,47^ these findings support the potential of icotrokinra as a targeted oral treatment option for active PsA.

## Data Availability

The data sharing policy of Johnson & Johnson is available at https://www.jnj.com/about-jnj/policies-and-positions/our-position-on-clinical-trial-data-transparency. As noted on this site, requests for access to the study data can be submitted through the Yale Open Data Access Project site at https://yoda.yale.edu.

## References

[1] Coates LC Helliwell PS . Psoriatic arthritis: state of the art review. Clin Med (Lond). 2017;17(1):65-70. doi:10.7861/clinmedicine.17-1-65. PMC6297592 28148584

[2] Ritchlin CT Colbert RA Gladman DD . Psoriatic arthritis. N Engl J Med. 2017;376(10):957-970. doi:10.1056/NEJMra1505557. 28273019

[3] Merola JF Tian H Patil D , et al. Incidence and prevalence of psoriatic arthritis in patients with psoriasis stratified by psoriasis disease severity: retrospective analysis of an electronic health records database in the United States. J Am Acad Dermatol. 2022;86(4):748-757. doi:10.1016/j.jaad.2021.09.019. 34547358

[4] Merola JF Chakravarty SD Choi O Chan D Gottlieb AB . A clinical review of structural damage in psoriatic arthritis for dermatologists: from pathogenesis to ongoing controversies. J Am Acad Dermatol. 2024;90(2):349-357. doi:10.1016/j.jaad.2023.10.021. 37852305

[5] James L Hailey LH Suribhatla R , et al. The impact of psoriatic arthritis on quality of life: a systematic review. Ther Adv Musculoskelet Dis. 2024;16:1759720X241295920. doi:10.1177/1759720X241295920. PMC11664531 39717741

[6] Krueger JG Eyerich K Kuchroo VK , et al. IL-23 past, present, and future: a roadmap to advancing IL-23 science and therapy. Front Immunol. 2024;15:1331217. doi:10.3389/fimmu.2024.1331217. PMC11056518 38686385

[7] Megna M Lauletta G D'Agostino M , et al. Biologics targeting IL-23 in moderate-to-severe psoriasis: lessons learned from real-world use. Expert Opin Biol Ther. 2025;25(7):711-729. doi:10.1080/14712598.2025.2519525. 40522209

[8] *STELARA (Ustekinumab) Injection, for Subcutaneous Use. Prescribing Information*. Janssen Biotech, Inc.; 2025. Accessed April 14, 2026. https://www.jnjlabels.com/package-insert/product-monograph/prescribing-information/STELARA-pi.pdf

[9] *TREMFYA^®^ (Guselkumab) Injection, for Subcutaneous Use. Prescribing Information*. Janssen Biotech, Inc.; 2025. Accessed April 14, 2026. https://www.jnjlabels.com/package-insert/product-monograph/prescribing-information/TREMFYA-pi.pdf

[10] *SKYRIZI^®^ (Risankizumab-rzaa) Injection, for Subcutaneous or Intravenous Use*. Prescribing information AbbVie; 2026. Accessed April 14, 2026. https://www.rxabbvie.com/pdf/skyrizi_pi.pdf

[11] *ILUMYA™ (tildrakizumab-asmn) injection, for subcutaneous use.* Prescribing Information. Sun Pharmaceutical Industries. Inc; 2025. Accessed April 14, 2026. https://www.ilumya.com/Sun_Pharma_ILUMYA_US_Prescribing_Information_.pdf

[12] McLenon J Rogers MAM . The fear of needles: a systematic review and meta-analysis. J Adv Nurs. 2019;75(1):30-42. doi:10.1111/jan.13818. 30109720

[13] Aletaha D Husni ME Merola JF , et al. Treatment mode preferences in psoriatic arthritis: a qualitative multi-country study. Patient Prefer Adherence. 2020;14:949-961. doi:10.2147/PPA.S242336. PMC7293411 32606613

[14] Fourie AM Cheng X Chang L , et al. JNJ-77242113, a highly potent, selective peptide targeting the IL-23 receptor, provides robust IL-23 pathway inhibition upon oral dosing in rats and humans. Sci Rep. 2024;14(1):17515. doi:10.1038/s41598-024-67371-5. PMC11289455 39080319

[15] ICOTYDE™ (Icotrokinra) Tablets, for Oral Use. Prescribing Information. Janssen Biotech, Inc; 2026. Accessed April 14, 2026. https://www.jnjlabels.com/package-insert/product-patient-information/ICOTYDE-medication-guide.pdf

[16] Bissonnette R Soung J Hebert AA , et al. Oral icotrokinra for plaque psoriasis in adults and adolescents. N Engl J Med. 2025;393(18):1784-1795. doi:10.1056/NEJMoa2504187. 41191940

[17] Stein Gold L Armstrong AW Bissonnette R , et al. Once-daily oral icotrokinra versus placebo and once-daily oral deucravacitinib in participants with moderate-to-severe plaque psoriasis (ICONIC-ADVANCE 1 & 2): two phase 3, randomised, placebo-controlled and active-comparator-controlled trials. Lancet. 2025;406(10510):1363-1374. doi:10.1016/S0140-6736(25)01576-4. 40976249

[18] Cauli A Gladman DD Mathieu A , et al. Patient global assessment in psoriatic arthritis: a multicenter GRAPPA and OMERACT study. J Rheumatol. 2011;38(5):898-903. doi:10.3899/jrheum.100857. 21324965

[19] Ye W Hackett S Vandevelde C Twigg S Helliwell PS Coates LC . Comparing the visual analog scale and the numerical rating scale in patient-reported outcomes in psoriatic arthritis. J Rheumatol. 2021;48(6):836-840. doi:10.3899/jrheum.200928. 33262305

[20] Langley RG Feldman SR Nyirady J van de Kerkhof P Papavassilis C . The 5-point Investigator’s Global Assessment (IGA) scale: a modified tool for evaluating plaque psoriasis severity in clinical trials. J Dermatolog Treat. 2015;26(1):23-31. doi:10.3109/09546634.2013.865009. 24354461

[21] Fredriksson T Pettersson U . Severe psoriasis–oral therapy with a new retinoid. Dermatologica (Basel). 1978;157(4):238-244. 10.1159/000250839 357213

[22] Cassell SE Bieber JD Rich P , et al. The modified nail psoriasis severity index: validation of an instrument to assess psoriatic nail involvement in patients with psoriatic arthritis. J Rheumatol. 2007;34(1):123-129. 17216680

[23] Blauvelt A Papp KA Griffiths CE , et al. Efficacy and safety of guselkumab, an anti-interleukin-23 monoclonal antibody, compared with adalimumab for the continuous treatment of patients with moderate to severe psoriasis: results from the phase III, double-blinded, placebo- and active comparator-controlled VOYAGE 1 trial. J Am Acad Dermatol. 2017;76(3):405-417. doi:10.1016/j.jaad.2016.11.041. 28057360

[24] Merola JF Bleakman AP Gottlieb AB , et al. The static Physician's global assessment of genitalia: a clinical outcome measure for the severity of genital psoriasis. J Drugs Dermatol. 2017;16(8):793-799. 28809994

[25] Leonardi C Langley RG Papp K , et al. Adalimumab for treatment of moderate to severe chronic plaque psoriasis of the hands and feet: efficacy and safety results from REACH, a randomized, placebo-controlled, double-blind trial. Arch Dermatol. 2011;147(4):429-436. doi:10.1001/archdermatol.2010.384. 21173304

[26] Feldman SR Mathias SD Schenkel B , et al. Development of a patient-reported outcome questionnaire for use in adults with moderate-to-severe plaque psoriasis: the psoriasis symptoms and signs diary. J Dermatol Dermatologic Surg. 2016;20(1):19-26.

[27] Gottlieb AB Kirby B Ryan C , et al. The development of the genital psoriasis sexual frequency questionnaire (GenPs-SFQ) to assess the impact of genital psoriasis on sexual health. Dermatol Ther. 2018;8(1):33-44. doi:10.1007/s13555-017-0212-3. PMC5825317 29204894

[28] Finlay AY Khan GK . Dermatology life quality index (DLQI)–a simple practical measure for routine clinical use. Clin Exp Dermatol. 1994;19(3):210-216. 10.1111/j.1365-2230.1994.tb01167.x 8033378

[29] Coates LC Corp N van der Windt DA Soriano ER Kavanaugh A . GRAPPA treatment recommendations: an update from the 2020 GRAPPA annual meeting. J Rheumatol. 2021;97:65-66. doi:10.3899/jrheum.201681. 34074672

[30] Chung CP Thompson JL Koch GG Amara I Strand V Pincus T . Are American College of Rheumatology 50% response criteria superior to 20% criteria in distinguishing active aggressive treatment in rheumatoid arthritis clinical trials reported since 1997? A meta-analysis of discriminant capacities. Ann Rheum Dis. 2006;65(12):1602-1607. doi:10.1136/ard.2005.048975. PMC1798472 16504992

[31] Felson DT Anderson JJ Boers M , et al. American College of Rheumatology. Preliminary definition of improvement in rheumatoid arthritis. Arthritis Rheum. 1995;38(6):727-735. doi:10.1002/art.1780380602. 7779114

[32] Queiro R Alonso S Pinto-Tasende JA . The nail in psoriatic arthritis: new insights into prognosis and treatment. Expert Opin Biol Ther. 2024;24(8):715-717. doi:10.1080/14712598.2024.2385020. 39044719

[33] Kaeley GS Eder L Aydin SZ Rich P Bakewell CJ . Nail psoriasis: diagnosis, assessment, treatment options, and unmet clinical needs. J Rheumatol. 2021;48(8):1208-1220. doi:10.3899/jrheum.201471. 33589557

[34] Singh JA Guyatt G Ogdie A , et al. Special article: 2018 American College of Rheumatology/National Psoriasis Foundation guideline for the treatment of psoriatic arthritis. Arthritis Rheumatol. 2019;71(1):5-32. doi:10.1002/art.40726. PMC8218333 30499246

[35] Gossec L Kerschbaumer A Ferreira RJO , et al. EULAR recommendations for the management of psoriatic arthritis with pharmacological therapies: 2023 update. Ann Rheum Dis. 2024;83(6):706-719. doi:10.1136/ard-2024-225531. PMC11103320 38499325

[36] Lynde C Riedl E Maul JT , et al. Comparative effectiveness of biologics across subgroups of patients with moderate-to-severe plaque psoriasis: results at week 12 from the PSoHO study in a real-world setting. Adv Ther. 2023;40(3):869-886. doi:10.1007/s12325-022-02379-9. PMC9988734 36515803

[37] Rech J Sticherling M Stoessel D Biermann MHC Haberle BM Reinhardt M . Psoriatic arthritis epidemiology, comorbid disease profiles and risk factors: results from a claims database analysis. Rheumatol Adv Pract. 2020;4(2):rkaa033. doi:10.1093/rap/rkaa033. PMC7585407 33134811

[38] Dopytalska K Sobolewski P Blaszczak A Szymanska E Walecka I . Psoriasis in special localizations. Reumatologia. 2018;56(6):392-398. doi:10.5114/reum.2018.80718. PMC6330687 30647487

[39] Strober B Ryan C van de Kerkhof P , et al. Recategorization of psoriasis severity: delphi consensus from the International Psoriasis Council. J Am Acad Dermatol. 2020;82(1):117-122. doi:10.1016/j.jaad.2019.08.026. 31425723

[40] Kristensen LE Keiserman M Papp K , et al. Efficacy and safety of risankizumab for active psoriatic arthritis: 24-week results from the randomised, double-blind, phase 3 KEEPsAKE 1 trial. Ann Rheum Dis. 2022;81(2):225-231. doi:10.1136/annrheumdis-2021-221019. PMC8762015 34911706

[41] Östör A Van den Bosch F Papp K , et al. Efficacy and safety of risankizumab for active psoriatic arthritis: 24-week results from the randomised, double-blind, phase 3 KEEPsAKE 2 trial. Ann Rheum Dis. 2022;81(3):351-358. doi:10.1136/annrheumdis-2021-221048. PMC8862056 34815219

[42] Deodhar A Helliwell PS Boehncke WH , et al. Guselkumab in patients with active psoriatic arthritis who were biologic-naïve or had previously received TNFα inhibitor treatment (DISCOVER-1): a double-blind, randomised, placebo-controlled phase 3 trial. Lancet. 2020;395(10230):1115-1125. doi:10.1016/s0140-6736(20)30265-8. 32178765

[43] Mease PJ Rahman P Gottlieb AB , et al. Guselkumab in biologic-naïve patients with active psoriatic arthritis (DISCOVER-2): a double-blind, randomised, placebo-controlled phase 3 trial. Lancet. A2020;395(10230):1126-1136. doi:10.1016/s0140-6736(20)30263-4. 32178766

[44] Nash P Mease PJ Kirkham B , et al. Secukinumab provides sustained improvement in nail psoriasis, signs and symptoms of psoriatic arthritis and low rate of radiographic progression in patients with concomitant nail involvement: 2-year results from the phase III FUTURE 5 study. Clin Exp Rheumatol. 2022;40(5):952-959. doi:10.55563/clinexprheumatol/3nuz51. 34494957

[45] McInnes IB Coates LC Mease PJ , et al. Sonelokimab, an IL-17A/IL-17F-inhibiting nanobody for active psoriatic arthritis: a randomized, placebo-controlled phase 2 trial. Nat Med. 2025;31(12):4160-4171. doi:10.1038/s41591-025-03971-6. PMC12705426 41053449

[46] Gooderham M Lain E Bissonnette R , et al. Targeted oral peptide icotrokinra for psoriasis involving high-impact sites. NEJM Evid. 2025;4(12):EVIDoa2500155. doi:10.1056/EVIDoa2500155. 41191932

[47] Bissonnette R Pinter A Ferris LK , et al. An oral interleukin-23-receptor antagonist peptide for plaque psoriasis. N Engl J Med. 2024;390(6):510-521. doi:10.1056/NEJMoa2308713. 38324484

[48] Lo Monaco M Mallaci Bocchio R Natoli G , et al. Critical importance of patient-reported outcomes for a comprehensive assessment of psoriatic arthritis patients. Front Med. 2023;10:1332432. doi:10.3389/fmed.2023.1332432. PMC10826607 38293304

